# AVM: A Manually Curated Database of Aerosol-transmitted Virus Mutations, Human Diseases, and Drugs

**DOI:** 10.1093/gpbjnl/qzae041

**Published:** 2024-06-04

**Authors:** Lan Mei, Yaopan Hou, Jiajun Zhou, Yetong Chang, Yuwei Liu, Di Wang, Yunpeng Zhang, Shangwei Ning, Xia Li

**Affiliations:** College of Bioinformatics Science and Technology, Harbin Medical University, Harbin 150081, China; College of Bioinformatics Science and Technology, Harbin Medical University, Harbin 150081, China; College of Bioinformatics Science and Technology, Harbin Medical University, Harbin 150081, China; College of Bioinformatics Science and Technology, Harbin Medical University, Harbin 150081, China; College of Bioinformatics Science and Technology, Harbin Medical University, Harbin 150081, China; College of Bioinformatics Science and Technology, Harbin Medical University, Harbin 150081, China; College of Bioinformatics Science and Technology, Harbin Medical University, Harbin 150081, China; College of Bioinformatics Science and Technology, Harbin Medical University, Harbin 150081, China; College of Bioinformatics Science and Technology, Harbin Medical University, Harbin 150081, China

**Keywords:** Aerosol, Viral mutation, Immune escape mutation, Human disease, Drug

## Abstract

Aerosol-transmitted viruses possess strong infectivity and can spread over long distances, earning the difficult-to-control title. They cause various human diseases and pose serious threats to human health. Mutations can increase the transmissibility and virulence of the strains, reducing the protection provided by vaccines and weakening the efficacy of antiviral drugs. In this study, we established a manually curated database (termed AVM) to store information on aerosol-transmitted viral mutations (VMs). The current version of the AVM contains 42,041 VMs (including 2613 immune escape mutations), 45 clinical information datasets, and 407 drugs/antibodies/vaccines. Additionally, we recorded 88 human diseases associated with viruses and found that the same virus can target multiple organs in the body, leading to diverse diseases. Furthermore, the AVM database offers a straightforward user interface for browsing, retrieving, and downloading information. This database is a comprehensive resource that can provide timely and valuable information on the transmission, treatment, and diseases caused by aerosol-transmitted viruses (http://www.bio-bigdata.center/AVM).

## Introduction

Aerosols are collections of solid or liquid particles suspended in gases (such as air) [1] that contain particles of various sizes. When droplets lose water in an air suspension, proteins and pathogens composing the droplet nuclei are transmitted through aerosols. A wide range of viruses can infect humans through aerosols, including severe acute respiratory syndrome coronavirus (SARS-CoV) [2], Middle East respiratory syndrome coronavirus (MERS-CoV) [3], H1N1 [4], severe acute respiratory syndrome coronavirus 2 (SARS-CoV-2) [5], and norovirus [6]. These viruses affect multiple organs, including the heart, kidney, liver, and small intestine, posing a serious threat to human health. However, integrated resources providing information on aerosol-transmitted viruses are scarce.

Droplet nuclei (< 5 μm) can remain suspended in the air for an extended period of time, dispersing over long distances (> 1 m) [7]. Body secretions and excretions containing viruses can be atomized in a number of ways to produce droplets or particles carrying the infectious pathogens, such as daily activities (*e.g.*, talking, breathing, and coughing) [8] and medical procedures (*e.g.*, endotracheal intubation, noninvasive ventilation, and bronchoscopy) [9,10], and the droplets are deposited on materials’ surfaces during human activities (*e.g.*, walking, cleaning the room, and opening the door) [11]. Infectious aerosols flood the environment continually.

When the respiratory tract or body surface mucosa is exposed to infectious aerosols containing viral particles, these pathogens may cause localized infections or destroy the host immune system to an extent wherein they can spread throughout the body, leading to the development of systemic illnesses. However, the exact mechanism of viral transmission through aerosols is not fully understood. Some viral mutations (VMs) notably affect disease development and progression by increasing or decreasing the pathogenicity or transmissibility of the virus [12,13]. For example, the mutation of P681H in the spike (S) protein of SARS-CoV-2, the pathogen of coronavirus disease 2019 (COVID-19), leads to a structural alteration that enhances the affinity of furin for the S protein. This alteration facilitates the penetration of the virus into the host cells, thereby augmenting its infectivity [14]. Mutants of the *S* gene with the R685H substitution exhibit a significant decrease in the production of infectious viruses, lowering the virus titer 12–100-fold compared with that of the wild-type (WT) virus. Additionally, the viral RNA levels of the *S* gene mutants are considerably lower than those of the WT virus [15]. Mutations in viral regions or genes may also increase the risk of disease progression and drug resistance [16,17]. A nonsynonymous change in N74S in the NA region of H1N1 results in reduced susceptibility to zanamivir, thereby increasing the risk of drug resistance [16]. However, most data are scattered in many independent studies, and there is currently no systematic compilation of mutations in aerosol-transmitted viruses.

In current public resources, professionally obtained knowledge on viruses is stored in freely accessible databases. The Global Initiative on Sharing All Influenza Data (GISAID) [18] is the world’s largest influenza and novel coronavirus data platform for the submission of novel coronavirus and influenza virus genome sequence information. The Immune Epitope Database and Analysis Resource (IEDB) [19] documents all experimental data on antibodies and T-cell epitopes in human and animal studies. The Virus Pathogen Database and Analysis Resource (ViPR) [20] is a comprehensive resource repository of data and analysis tools that caters to numerous virus families. The PathoSystems Resource Integration Center (PATRIC) [21] is dedicated to the development and integration of a drug-resistance gene database system. GenBank [22] is a DNA sequence database developed by the National Center for Biotechnology Information (NCBI). However, existing databases lack compilation and integration of pathogenic VMs found in aerosol-transmitted viruses. Databases providing systematic evaluation and functional annotation of VMs are especially needed because of increased concerns about viruses that cause human diseases in the wake of the COVID-19 outbreak.

**Figure 1:**
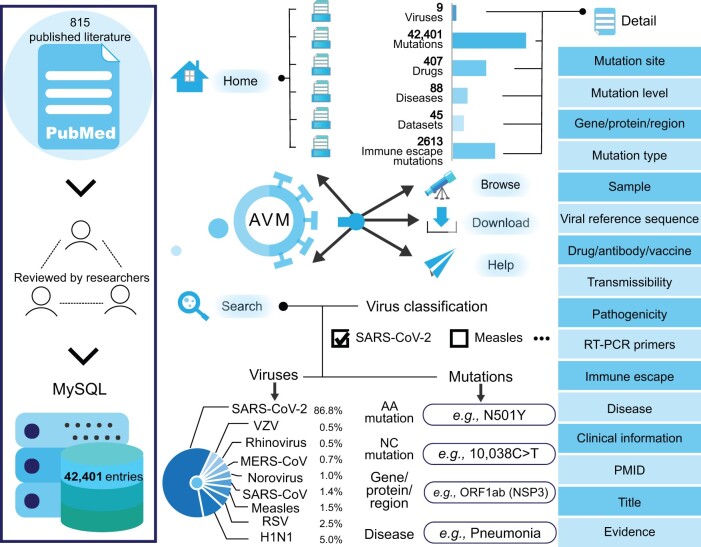
Data source and the structure of AVM SARS-CoV, severe acute respiratory syndrome coronavirus; MERS-CoV, Middle East respiratory syndrome coronavirus; SARS-CoV-2, severe acute respiratory syndrome coronavirus; VZV, varicella-zoster virus; RSV, respiratory syncytial virus; AA, amino acid; NC, nucleotide; PMID, PubMed Unique Identifier; RT-PCR, reverse transcription polymerase chain reaction.

Therefore, we constructed the AVM, a manually crafted database of human viruses transmitted via aerosols, to provide a comprehensive and professional resource for aerosol-transmitted viruses (**Figure 1**). The AVM includes information on VMs, drug information, diseases, clinical data, and immune escape. This database also offers experimentally validated data regarding the effects of VMs on protein function. This is the most comprehensive database for aerosol-transmitted viruses. The repository can be accessed at http://www.bio-bigdata.center/AVM.

## Data collection and processing

To assess the evidence of virus transmission through aerosols, we utilized the criteria outlined by Jones and Brosseau [23]. According to these criteria, aerosol transmission is considered plausible under the following conditions: (1) aerosols containing the pathogen are produced by an infected individual; (2) the pathogen can remain active in the environment for a certain period; and (3) the aerosol has the potential to reach and infect the target tissues. We assigned a value to each condition based on the strength of the evidence (1 = weak, 2 = moderate, and 3 = strong) and summed these values across the three conditions. We then assigned a score of 6 or higher to indicate the possibility of aerosol transmission. In the current version, the database incorporates nine viruses, namely, MERS-CoV, respiratory syncytial virus (RSV), measles virus, norovirus, rhinovirus, varicella-zoster virus (VZV), SARS-CoV-2, SARS-CoV, and H1N1. They all meet the criteria proposed by Jones and Brosseau [23] and had a score between 8 and 9 (maximum score: 9) (Table S1).

To maintain the quality of the AVM database in the collection process, each entry was meticulously gathered through a series of steps, which were employed for the construction of the database of human disease-related VMs, integration sites, and *cis*-effects [24], the comprehensive resource for SARS-CoV-2 immune escape variants [25], and the manually curated database of experimentally supported associations among microRNAs (miRNAs), single-nucleotide polymorphisms (SNPs), and human diseases [26].

First, we searched the PubMed database [27] using the keywords “human H1N1 mutation” and “human H1N1 variant”. The same approach was followed for all of the other viruses (*e.g.*, “human measles virus mutation” and “human measles virus variant”, “human norovirus mutation” and “human norovirus variant”, and “human RSV mutation” and “human RSV variant”) to filter the literature by matching keywords. We extracted key information by downloading all of the literature on VMs from peer-reviewed publications and preprints.

Second, we manually extracted information on VMs from academic articles. All the selected articles were evaluated by at least two researchers. During this stage, we recorded mutation sites, mutation levels (*e.g.*, amino acid level and nucleotide level), mutation types (*e.g.*, synonymous mutation and nonsynonymous mutation), viral genes/proteins/regions, NCBI IDs, virus genotypes/subtypes/clades (genotypes or subtypes mentioned in the literature), drugs/antibodies/vaccines (mentioned in the literature), virus-associated diseases, clinical information available in the literature, virus samples (specimens derived from humans or cell lines), country/region (geographical distribution of the mutation), virus variants (virus subspecies), reference sequence, transmissibility (effect of VMs on viral transmissibility, *i.e.*, promote or hinder), pathogenicity (effect of VMs on viral pathogenicity, *i.e.*, increase or decrease), use of the reverse transcription polymerase chain reaction (RT-PCR) primer probes (yes or no), immune escape (VMs related to immune escape), evidence (*i.e.*, a sentence containing information about the VM in the literature), protein introduction (protein name, UniProt ID, protein length, and protein description), and literature information (PubMed ID, year of publication, journal name, and paper title).

Additionally, we searched the PubMed database for “H1N1 human disease” to obtain information on the human diseases related to H1N1, and the same approach was followed for the other viruses (*e.g.*, measles virus human disease, norovirus human disease, and RSV human disease). The modes of transmission, pathogenicity, and immune escape altered by VMs were obtained from high-quality studies with robust experimental evidence. Finally, we used a standardized naming scheme, the Human Genome Variation Society (HGVS) rules, to annotate each mutation.

## Database content and construction

**Figure 2:**
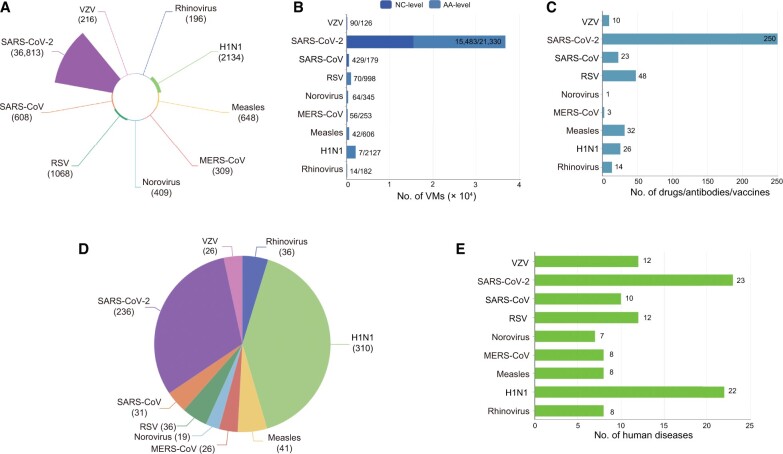
Information on AVM **A**. Number of mutation entries for diverse viruses. **B**. Number of NC mutations and AA mutations for diverse viruses. The first number represents the count of NC-level mutations, and the second number represents the count of AA-level mutations. The two numbers are separated by “/”. The sum of the two numbers is the total count of mutations of this virus. **C**. Number of drugs, antibodies, or vaccines for diverse viruses. **D**. Number of articles in PubMed for diverse viruses. **E**. Number of human diseases caused by diverse viruses.

After completing this process, more than 810 articles were systematically reviewed, and a total of 42,041 mutation entries (including 2613 immune escape mutations), 45 clinical information datasets, and 407 drugs/antibodies/vaccines were manually curated (as of October 2, 2022). Additionally, we recorded data on 88 human diseases (**Figure 2**; **Table 1**). Moreover, we provided a brief description of each protein involved in mutations, including the protein name, protein length, hyperlinks to UniProt [28], and the structure and function of each protein. Furthermore, we provided a brief description of the mechanism by which a mutation leads to changes in the transmissibility and/or pathogenicity of the virus from the original study.

**Table 1:** Statistics for the mutation entries in the AVM database

Virus	No. of mutation entries	No. of immune escape mutations	No. of drugs/antibodies/vaccines	No. of datasets	No. of diseases
SARS-CoV-2	36,813	2316	250	9	23
RSV	1068	58	48	5	12
Measles virus	648	99	32	1	8
Norovirus	409	0	1	1	7
Rhinovirus	196	16	14	0	8
VZV	216	0	10	3	12
H1N1	2134	57	26	23	22
SARS-CoV	608	34	23	0	10
MERS-CoV	309	33	3	3	8
Sum	42,401	2613	407	45	88

*Note*: SARS-CoV, severe acute respiratory syndrome coronavirus; MERS-CoV, Middle East respiratory syndrome coronavirus; SARS-CoV-2, severe acute respiratory syndrome coronavirus; VZV, varicella-zoster virus; RSV, respiratory syncytial virus.

Ultimately, all AVM data were saved and administered using MySQL (v8.0.17). Java Server Page (JSP) was used to construct the web interface, and Java (v1.8.0_291) was employed to code the data processing programs. The structure of the web services was achieved using Apache Tomcat as the underlying framework. The AVM database can be freely accessed at http://www.bio-bigdata.center/AVM.

## User interface

**Figure 3:**
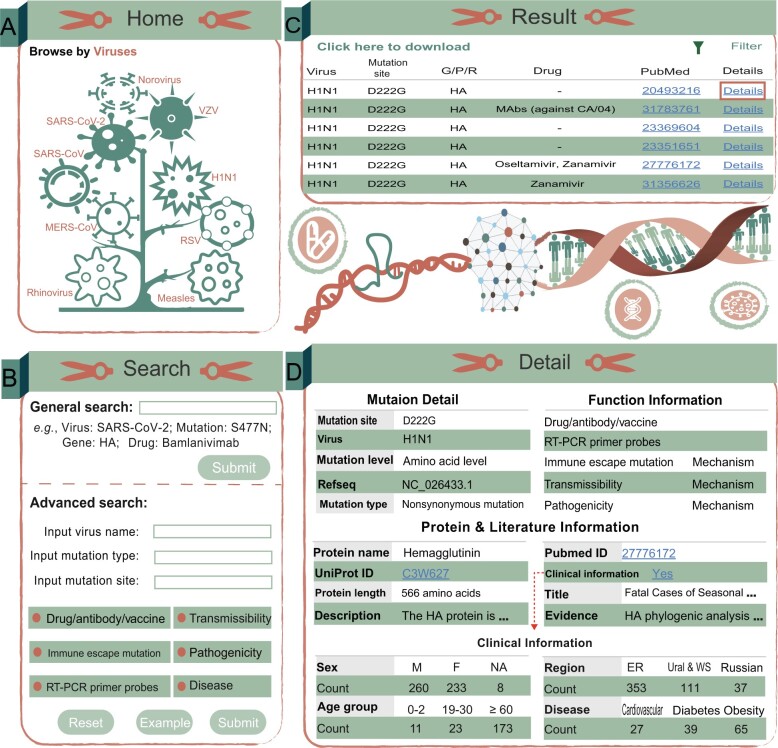
A schematic workflow of AVM **A**. Users can click on virus images to browse mutation information related to this virus. **B**. “Search” interface in a fuzzy and advanced manner. **C**. Results of the “Home” or “Search” page. **D**. Detailed information and clinical information of the AVM entries. ER, European Russia; WS, Western Siberia.

The AVM renders an easy-to-use web interface that allows users to surf, search, and acquire data (**Figure 3**). The database presents a concise introduction to viral genes and diseases caused by each virus on its respective interface, enabling users to swiftly verify their hypotheses regarding the VMs.

The statistics on the number of mutation entries for each virus are elaborated in the “Home” page. Additionally, we provided a fuzzy search function. Users can click on a virus image and see relevant virus information; they can also search for specific viruses, VMs, genes, or drugs. Users can consult a mutation site with a mutated gene/protein/region, drugs, and literature PubMed Unique Identifier (PMID). The detailed VM page contains mutation annotations, protein descriptions, and literature information.

In the “Search” page, AVM allows users to search by amino acid mutation, nucleotide mutation, virus name, gene name, and drug. AVM also offers an advanced search that allows users to screen for drugs/antibodies/vaccines mentioned in the literature, viral transmissibility, viral pathogenicity, RT-PCR probe-binding sites, immune escape mutations, and diseases.

The “Browse” page visually demonstrates the number of mutations of each virus in the database, the number of reference articles, the number of nucleotide and amino acid mutations, the number of related drugs and antibodies, and the diseases caused by the viruses. The users can download all the data from AVM on the “Download” page. The “Help” page provides a detailed tutorial explaining how the users can utilize AVM effectively.

## Database application

### Cases of transmissibility and pathogenicity

Owing to the accumulation of experimentally demonstrated functional annotations of VMs associated with transmissibility and pathogenicity, AVM could offer mechanistic notions and an experimental basis for future research. As an illustration, by searching AVM for “A570D (PMID: 34385690)”, a mutation in the S protein of SARS-CoV-2, we found that A570D hindered the spread of the virus. A description of the transmission mechanism showed that A570D-mediated salt bridges can act as pedals in a pedal-bin-like mechanism, controlling the motion of the receptor-binding domain. This modulation was expected to affect ACE2 binding and viral infection. For another example, we searched AVM for “L452R (PMID: 34171266)” in the *S* gene of SARS-CoV-2, which promotes virus transmission. The increased viral infectivity resulting from the L452R substitution was strongly associated with an intensified electrostatic interaction with ACE2, likely because residue 452 is situated near a cluster of negatively charged ACE2 residues.

Moreover, we searched AVM for “F49A (PMID: 33999154)” in ORF1ab (NSP7) of SARS-CoV-2, which decreased the pathogenicity of the virus. The “Description mechanism” displayed that the mutations on the heterodimeric interface I of NSP7 (NSP7–F49A and NSP7–L56A) and NSP8 (NSP8–F92A) resulted in enhanced NSP8 dimerization, leading to a decrease in the formation of the NSP7–NSP8 heterotetramer. This indicates that these mutations likely disrupt the association between NSP7 and NSP8. AVM contains a large amount of mutation information about virus transmissibility and pathogenesis verified by experiments, which is beneficial for studying virus transmission mechanism and pathogenicity and for further exploring the regulation mechanism of the virus, thus making it possible to produce specific antiviral drugs.

### Cases of immune escape and drug resistance

Moreover, AVM provides data on immune escape and drug resistance sites, along with information on mutations resistant to antibodies/vaccines/drugs. These data could help professionals choose more scientific and rational treatment strategies while also enabling patients to achieve the best therapeutic benefits. For example, by searching AVM using “bamlanivimab resistant” as input in a general search, we found 32 entries including A67V, D796Y, E484A, and E484K to be resistant to bamlanivimab, a commonly used monoclonal antibody drug for SARS-CoV-2 virus. Administration of bamlanivimab should be avoided in clinical practice for patients with these mutations, and treatment strategies should consider dual antibody therapy or other monoclonal antibody therapies. As another example, we searched for “P653L (PMID: 34061908)”, a mutation in PA of H1N1. The results revealed that P653L was resistant to favipiravir. By searching AVM for “favipiravir resistant” as input in a general search, another mutation of K229R in PB1 appeared, also resistant to favipiravir. Furthermore, AVM will likely facilitate the design of vaccines and neutralizing monoclonal antibodies and guide the research and development of drugs. AVM also includes several clinical datasets; hence, senior users can conveniently access relevant virus data and conduct more in-depth analyses independently.

## Conclusion and future direction

Recently, with the increasing popularity of sequencing technology, extensive biological data on viruses have been generated. Many VMs, especially in key viral genes or proteins that lead to disease progression or drug resistance, have been found. Thus, we created AVM, a database of aerosol-transmitted viruses, which provides a comprehensive resource on VMs, drugs, and aerosol-transmitted human diseases. The AVM database integrates experimental data on the aerosol transmission of human pathogenic viruses, providing the necessary resources to comprehend and further study viral transmission, especially in different viruses.

AVM will be further maintained and updated as VM data accumulates. We will incorporate new analysis tools to enhance the practicability of AVM, add an upload module to empower users to submit VMs of interest, and augment the Representational State Transfer Application Programming Interface (RESTful API) to keep pace with the research. We hope that AVM will aid the biomedical community in obtaining an all-encompassing comprehension of research studies on viruses, and expedite the development of prophylactics and therapeutics for aerosol-transmitted viruses.

## Supplementary Material

qzae041_Supplementary_Data

## Data Availability

The AVM database can be accessed at http://www.bio-bigdata.center/AVM.

## CRediT author statement

**Lan Mei:** Methodology, Validation, Data curation, Visualization, Investigation, Writing – original draft. **Yaopan Hou:** Software, Visualization. **Jiajun Zhou:** Data curation, Investigation. **Yetong Chang:** Investigation. **Yuwei Liu:** Investigation. **Di Wang:** Investigation. **Yunpeng Zhang:** Supervision. **Shangwei Ning:** Supervision, Writing – review & editing, Funding acquisition. **Xia Li:** Conceptualization, Project administration. All authors have read and approved the final manuscript.
