# Supplementary material for: AVM: A Manually Curated Database of Aerosol-transmitted Virus Mutations, Human Diseases, and Drugs
Source: Genomics Proteomics Bioinformatics. 2024 Jun 4;22(3):qzae041. doi: 10.1093/gpbjnl/qzae041 (PMC12016557; doi:10.1093/gpbjnl/qzae041)
Supplement: qzae041_Supplementary_Data [file qzae041_supplementary_data.zip › Supplementary material captions.docx]

**Supplementary** **material**

**Table S1 Evidence of virus aerosol transmission**
