# Supplementary material for: AVM: A Manually Curated Database of Aerosol-transmitted Virus Mutations, Human Diseases, and Drugs
Source: Genomics Proteomics Bioinformatics. 2024 Jun 4;22(3):qzae041. doi: 10.1093/gpbjnl/qzae041 (PMC12016557; doi:10.1093/gpbjnl/qzae041)
Supplement: qzae041_Supplementary_Data [file qzae041_supplementary_data.zip › Table S1-done.docx]

**Table S1 Evidence of virus aerosol transmission­**

| **Virus** | **Aerosol generation** | **Quality**  **score** | **Viability in environment** | **Quality**  **score** | **Access to target tissue** | **Quality score** | **Aerosol score** | **Ref.** | |
| --- | --- | --- | --- | --- | --- | --- | --- | --- | --- |
| RSV | A: Aerosols containing the pathogen are released from an infected person | 3 | A: The pathogen can survive outside a host and remain suspended in air for a significant period of time | 3 | B: Experimental infection by aerosol approach has been demonstrated in a mouse animal model | 2 | 8 | [1]  [2] | |
| VZV | A: Aerosols containing the pathogen are emitted from an infectious person | 3 | A: In the previously occupied patient ward, the pathogen can be detected within 24 hours | 3 | A: Aerosol transmission of infectious pathogens has been proven | 3 | 9 | [3]  [4] | |
| MERS-  CoV | A: Aerosols containing the pathogen are found in the air of isolation wards | 3 | A: The atomized virus has a long survival time in the environment and is stable | 3 | B: Target tissues have been identifieda in human infection experiments through in vitro studies and non-aerosol routes, and transmission through aerosols seems plausible | 2 | 8 | [5]  [6]  [7] | |
| SARS-  CoV | A: Patients release aerosols containing the pathogen | 3 | A: Epidemiological evidence indicates transmission of aerosols over long distance | 3 | A: Experimental human infection via aerosol route has been confirmed | 3 | 9 | [8]  [9] | |
| SARS-  CoV-2 | A: Infected individuals release aerosols containing the pathogen | 3 | A: The pathogen remains infectious after surviving in aerosols around laboratory media for up to 16 hours | 3 | B: Experimental infection by aerosol approach has been demonstrated in hamster models | 2 | 8 | [10]  [11]  [12] | |
| Norovirus | A: Air samples taken from the patient's room and toilet are positive for the virus, suggesting viral atomization in the patient's vomit | 3 | A: The pathogen can survive in the air for a certain period of time under suitable environmental conditions. Furthermore, in vitro aerosol studies have demonstrated that the pathogen retains the infectiousness and integrity | 3 | B: Target tissues have been identified in human infection experiments via the non-aerosol approach. Alternatively, infection can be reasonably achieved through aerosol in vitro | 2 | 8 | [13]  [14]  [15] | |
| H1N1 | A: The virus is emitted as an aerosol from infected individuals | 3 | B: The pathogen remains infectious after surviving in aerosols around patients for several hours | 2 | A: Experimental human infection via aerosol route has been confirmed | 3 | 8 | [16]  [17]  [18] | |
| Rhinovirus | A: Aerosols containing the pathogen are released from infected individuals | 3 | A: Under specific air conditions, the pathogen survives for several hours when cultured in laboratory media or fluids from the body | 3 | A: Experimental human infection via aerosol route has been verified | 3 | 9 | [19]  [20]  [21] | |
| Measles | A: Aerosols containing the pathogen are released from infected individuals | 3 | A: Under certain ambient conditions, the pathogen can remain viable for several hours in the air, or when cultured in laboratory media or bodily fluids | 3 | B: Experimental infection by aerosol approach has been demonstrated in bovine animal models | 2 | 8 | [22]  [23]  [24] | |
| *Note*: A, B, and C represent the quality of evidence. A means strong with score 3; B means moderate score with 2; C means weak score with 1. Aerosol score is the sum of the three evidence scores | | | | | | | | |  |

**References**

[1] Aintablian N, Walpita P, Sawyer MH. Detection of Bordetella pertussis and respiratory synctial virus in air samples from hospital rooms. Infect Control Hosp Epidemiol 1998;19:918−23.

[2] Lindsley WG, Blachere FM, Davis KA, Pearce TA, Fisher MA, Khakoo R, et al. Distribution of airborne influenza virus and respiratory syncytial virus in an urgent care medical clinic. Clin Infect Dis 2010;50:693−8.

[3] Sawyer MH, Chamberlin CJ, Wu YN, Aintablian N, Wallace MR. Detection of varicella-zoster virus DNA in air samples from hospital rooms. J Infect Dis 1994;169:91−4.

[4] Gustafson TL, Lavely GB, Brawner ER, Jr., Hutcheson RH, Jr., Wright PF, Schaffner W. An outbreak of airborne nosocomial varicella. Pediatrics 1982;70:550−6.

[5] Kim SH, Chang SY, Sung M, Park JH, Bin Kim H, Lee H, et al. Extensive viable Middle East respiratory syndrome (MERS) coronavirus contamination in air and surrounding environment in MERS Isolation Wards. Clin Infect Dis 2016;63:363−9.

[6] van Doremalen N, Bushmaker T, Munster VJ. Stability of Middle East respiratory syndrome coronavirus (MERS-CoV) under different environmental conditions. Euro Surveill 2013;18:20590.

[7] Ki M. 2015 MERS outbreak in Korea: hospital-to-hospital transmission. Epidemiol Health 2015;37:e2015033.

[8] Tsai YH, Wan GH, Wu YK, Tsao KC. Airborne severe acute respiratory syndrome coronavirus concentrations in a negative-pressure isolation room. Infect Control Hosp Epidemiol 2006;27:523−5.

[9] Yu IT, Li Y, Wong TW, Tam W, Chan AT, Lee JH, et al. Evidence of airborne transmission of the severe acute respiratory syndrome virus. N Engl J Med 2004;350:1731−9.

[10] Liu Y, Ning Z, Chen Y, Guo M, Liu Y, Gali NK, et al. Aerodynamic analysis of SARS-CoV-2 in two Wuhan hospitals. Nature 2020;582:557−60.

[11] Fears AC, Klimstra WB, Duprex P, Hartman A, Weaver SC, Plante KS, et al. Persistence of severe acute respiratory syndrome coronavirus 2 in aerosol suspensions. Emerg Infect Dis 2020;26:2168−71.

[12] Sia SF, Yan LM, Chin AWH, Fung K, Choy KT, Wong AYL, et al. Pathogenesis and transmission of SARS-CoV-2 in golden hamsters. Nature 2020;583:834−8.

[13] Alsved M, Fraenkel CJ, Bohgard M, Widell A, Söderlund-Strand A, Lanbeck P, et al. Sources of airborne norovirus in hospital outbreaks. Clin Infect Dis 2020;70:2023−8.

[14] Bonifait L, Charlebois R, Vimont A, Turgeon N, Veillette M, Longtin Y, et al. Detection and quantification of airborne norovirus during outbreaks in healthcare facilities. Clin Infect Dis 2015;61:299−304.

[15] Atmar RL, Opekun AR, Gilger MA, Estes MK, Crawford SE, Neill FH, et al. Determination of the 50% human infectious dose for Norwalk virus. J Infect Dis 2014;209:1016−22.

[16] Lindsley WG, Noti JD, Blachere FM, Thewlis RE, Martin SB, Othumpangat S, et al. Viable influenza A virus in airborne particles from human coughs. J Occup Environ Hyg 2015;12:107−13.

[17] Bischoff WE, Swett K, Leng I, Peters TR. Exposure to influenza virus aerosols during routine patient care. J Infect Dis 2013;207:1037−46.

[18] Hao XY, Li FD, Lv Q, Xu YF, Han YL, Gao H. Establishment of BALB/C mouse models of influenza A H1N1 aerosol inhalation. J Med Virol 2019;91:1918−29.

[19] Huynh KN, Oliver BG, Stelzer S, Rawlinson WD, Tovey ER. A new method for sampling and detection of exhaled respiratory virus aerosols. Clin Infect Dis 2008;46:93−5.

[20] Karim YG, Ijaz MK, Sattar SA, Johnson-Lussenburg CM. Effect of relative humidity on the airborne survival of rhinovirus-14. Can J Microbiol 1985;31:1058−61.

[21] Couch RB, Cate TR, Douglas RG, Jr., Gerone PJ, Knight V. Effect of route of inoculation on experimental respiratory viral disease in volunteers and evidence for airborne transmission. Bacteriol Rev 1966;30:517−29.

[22] Bloch AB, Orenstein WA, Ewing WM, Spain WH, Mallison GF, Herrmann KL, et al. Measles outbreak in a pediatric practice: airborne transmission in an office setting. Pediatrics 1985;75:676−83.

[23] Ehresmann KR, Hedberg CW, Grimm MB, Norton CA, MacDonald KL, Osterholm MT. An outbreak of measles at an international sporting event with airborne transmission in a domed stadium. J Infect Dis 1995;171:679−83.

[24] Lemon K, de Vries RD, Mesman AW, McQuaid S, van Amerongen G, Yüksel S, et al. Early target cells of measles virus after aerosol infection of non-human primates. PLoS Pathog 2011;7:e1001263.
